# Deletion of sorting nexin 27 suppresses proliferation in highly aggressive breast cancer MDA-MB-231 cells in vitro and in vivo

**DOI:** 10.1186/s12885-019-5769-z

**Published:** 2019-06-10

**Authors:** Jilei Zhang, Kendy Li, Yongguo Zhang, Rong Lu, Shaoping Wu, Jingrong Tang, Yinglin Xia, Jun Sun

**Affiliations:** 1 Division of Gastroenterology and Hepatology, Medicine, University of Illinois at Chicago, 840 S Wood Street, Room 704 CSB, MC716, Chicago, IL 60612 USA; 2 Liberal Arts & Human Sciences, Virginia Polytechnic Institute and State University, Blacksburg, Virginia, USA; 3 Department of Biochemistry, Rush University, Chicago, IL USA; 4 Section of Inflammation and Cardiometabolic Diseases, National Heart, Lung, and Blood Institute (NHLBI), National Institutes of Health, Bethesda, MD USA

**Keywords:** Epithelial-mesenchymal transition, Cell adhesion, Cell junctions, Breast cancer, Sorting nexin 27, Proliferation

## Abstract

**Background:**

Sorting Nexin 27 (SNX27) belongs to a family of sortin nexins and possesses a unique binding domain at the C-terminus which mediates protein-protein interaction in intracellular trafficking, membrane remodeling, organelle motility, and tight junctions. However, its role in cancer development, especially in vivo, remains largely unknown.

**Methods:**

We have generated a stable SNX27 knockdown clone in a highly aggressive breast cancer cell line MDA-MB-231 using an inducible lentiviral shRNA system. Cell migration and proliferation of SNX27 knockdown (KD) cells were compared with wild-type (WT) cells by MTT and wound healing assay, respectively. The differences in colony formation between SNX27-KD and WT cells were detected by soft agar culture and matrigel 3D culture. Furthermore, tumor growth was examined in a xenograft nude mouse model using SNX27-KD and WT MDA-MB-231 cells. The critical EMT (epithelial-mesenchymal transition) regulators were examined in vitro and in vivo.

**Results:**

The wound healing assay showed that SNX27 knockdown significantly decreased cell motility and proliferation. Colony formation in soft agar showed that the SNX27 knockdown cells formed significantly fewer and smaller colonies than the parental MDA-MB-231 cells. Western blots and immunostaining showed that knockdown of SNX27 led to increased expression of E-cadherin and β-catenin proteins, which facilitate adhesion formation and reverse EMT. EMT is a cellular program that allows polarized, immotile epithelial cells to convert to motile mesenchymal cells, promoting carcinoma invasion. The expression levels of Vimentin, the transcription factor of EMT, and tight junction protein Claudin-5, were significantly diminished in the SNX27 knockdown cells. The expression of PCNA, the cell proliferation marker, was increased in SNX27-KD cells transfected with E-cadherin siRNA. In a xenograft nude mouse model, we found that knockdown of SNX27 significantly inhibited tumor growth. The tumors from mice with SNX27-KD cells showed less proliferation compared to tumors from mice injected with wildtype cells. The increase in E-cadherin and β-catenin and decrease in Vimentin and Claudin-5 were observed in tumors of mice injected with SNX27-KD cells.

**Conclusions:**

Our data have demonstrated that SNX27 plays a crucial role in tumor growth in vitro and in vivo.

## Background

Sorting Nexins (SNXs) are peripheral membrane proteins. They are grouped with the subfamily of the Phox-homology (PX) domain family, based on the presence of SNX-PX domain [1, 2]. SNXs rescue transmembrane proteins from the lysosomal degradative pathway and facilitate their recycling to other cellular compartments as well as play roles in membrane trafficking, cell signaling, membrane remodeling, organelle motility, ion channel regulation and receptor recycling [1, 3]. The sorting nexin 27 (SNX27), which contains a PSD95, Dlg1, ZO-1 (PDZ)-binding motif, promotes recycling of internalized transmembrane proteins from endosomes to the plasma membrane by linking PDZ-dependent cargo recognition to retromer-mediated transport or regulate of endosome-to-plasma membrane recycling of transmembrane [4, 5]. More than 100 cell surface proteins require SNX27-retromer, linking to prevent lysosomal degradation and maintain surface levels [5–8].

Breast cancer is the most common cancer in women. Approximately 90% of the deaths in breast cancer are caused by local invasion and distant metastasis [9–11]. Recent studies have revealed mechanisms through which multiple cancer cell and stromal cell subpopulations interact, including paracrine signaling, direct cell-cell adhesion, and remodeling of the extracellular matrix [10]. Three cell interaction mechanisms have emerged to explain how breast tumors become invasive: EMT, collective invasion, and the macrophage-tumor cell feedback loop [10, 11]. EMT is a reversible and transient process which enables epithelial tumor cells to gain access to the vasculature and the formation of distant metastasis [12]. Multiple genes and proteins (e.g., Cadherins, β-catenin, and Vimentin) play crucial roles in EMT, thus serving as possible markers in the assessment of EMT. Cell-cell adhesion is mediated by a variety of membrane proteins, such as classical E or N-cadherins, claudins, and β-catenin [13–15]. Classical cadherins are essential in initiating cell-cell contacts. Moreover, E-cadherin is a tumor suppressor and used as a prognostic marker for breast cancer treatment [15, 16]. Previous studies have reported that SNX27 regulates focal adhesions and cell motility [17] and polarizes to the apical membrane during NK cell migration [9]. SNX27 also interacts with Frizzled (Fzd) receptors to regulate the endocytosis and stability of Fzds and consequently mediates canonical Wnt/β-catenin signalling [18]. However, whether SNX27 affects breast cancer has not been explored.

In the current study, we hypothesized that reduction of SNX27 in cancer cells suppresses proliferation. We compared the SNX27 levels in various breast cancer cell lines and selected a highly aggressive breast cancer cell line MDA-MB-231 with the highest level of SNX27 protein expression. We then generated a stable SNX27 knockdown clone in the MDA-MB-231 cells using an inducible lentiviral shRNA system. We showed that knockdown of SNX27 could reduce tumor cell migration, enhance the cell-cell contacts, and suppress cancer growth. Thus, our data suggest that SNX27 plays a critical role in cancer development.

## Methods

### Materials

The monoclonal antibody to human SNX27 (N-terminal amino acids 1–267) was purchased from Abcam (Cambridge, MA). Anti-GAPDH monoclonal antibody was purchased from Aldrich (St. Louis, MO). A whole set of the EMT Antibody Sampler Kit was purchased from Cell Signalling (Danvers, MA), including monoclonal antibodies to human β-catenin, E-cadherin, N-Cadherin, Vimentin and TCF-β/ZEB1 raised in rabbit. Anti-human β-catenin, anti-human E-cadherin and anti-human Claudin-5 from BD Transduction (San Jose, CA), and anti-human β-actin from Sigma-Aldrich (St. Louis, MO) were used to western blot. Mouse monoclonal c-Myc antibody purchased from Santa Cruz Biotechnology (Dallas, TX) was used for the c-Myc expression detection. The expression of PCNA was detected using mouse monoclonal PCNA antibody from Cell Signalling (Danvers, MA). All chemicals were purchased from Sigma-Aldrich unless otherwise stated. The highly aggressive breast cancer cell lines, including MDA-MB-231, MDA-MB-435, MDA-MB-436, MCF-7A and MX1, were provided by Dr. Chu-Xia Deng’s lab at National Institute of Diabetes and Digestive and Kidney Diseases (NIDDK), National Institutes of Health. (NIH). The SNX27 KD cell line was provided by Dr. Martin Playford at National Institutes of Health. MCF-10A (ATCC CRL-10317) and MCF-10-2A (ATCC CRL-10781) were purchased from ATCC (Manassas, VA). All the cell lines were tested for mycoplasma contamination before being performed in this study using LookOut Mycoplasma PCR Detection Kit (Sigma-Aldrich, St. Louis, MO).

### Cell culture and Knock-down of SNX27 in MDA-MB-231 cells

Cells were maintained in high glucose, Dulbecco’s modified Eagle’s medium (Invitrogen, Carlsbad, CA) supplemented with 10% fetal bovine serum (Invitrogen) in humidified chambers with 5% CO_2_ at 37 °C. MCF-10AH1 cells were maintained in high glucose, Dulbecco’s modified Eagle’s medium (Invitrogen) supplemented with 10% normal horse serum (Invitrogen, Carlsbad, CA). Stable shRNA expressing cells were generated using an inducible lentiviral shRNA system (pINDUCER10) and selected with Puromycin [15]. Clone 5 of shRNA of SNX27 has been used to target human SNX27 with the sequence TGGTCTGTAGTACTGTTCT. These sequences were subcloned from pGIPZ to pINDUCER10 with MluI and XhoI digests. An identical method was used to prepare control (firefly luciferase) lentivirus. Lentiviral supernatants were generated by transient transfection of MBA-MD-231 cells using FuGENE HD [17]. Several clones were isolated and the expression of SNX27 was confirmed by western blot analysis with anti-SNX27 antibody. shRNA expression on puromycin-resistant clones were routinely induced with 1.5 μg/ml of doxycycline for 72–96 h prior to experimental analysis. The SNX27-KD clone could be maintained in selection medium with 2.5 μg/ml puromycin and 1.5 μg/ml doxycycline for several weeks.

### Western blot analysis

Cells were rinsed twice with ice-cold HBSS, lysed in 250 μl of ice-cold binding buffer 150 (BB150: 50 mM Tris, pH 7.6, 150 mM NaCl, 0.2% CHAPS, 10 mM EDTA) plus Complete TM protease inhibitor mixture (Roche Applied Science, Penzberg, Germany). The lysate was purified by centrifugation at 15,000 x g for 10 min at 4 °C to clear the cellular debris. Total protein was quantified using the bicinchoninic acid assay (Pierce Biotechnology, Rockford, IL). Equal amount of protein was loaded on NuPAGE® Novex® 4–12% Bis-Tris in NuPAGE® MOPS SDS Running Buffer (Invitrogen, Carlsbad, CA), and transferred to nitrocellulose membrane by iBlot® - Western Blotting System from Invitrogen. Immuno-detection was performed by blocking the membranes for 1 h in Blocking buffer (Licor, Lincoln NE). SNX27 was detected by primary anti-human SNX27 raised in rabbit (1:2000) followed by anti-rabbit secondary antibody conjugated to IRdye 780 nm (Licor, Lincoln, NE) at 1:10,000 dilution. The proteins associated with EMT including were with primary antibody raised in rabbit (1:1000) and followed by secondary antibodies conjugated to IRdye 780 nm at 1:10,000 dilution. The image was scanned and analyzed by Licor odyssey (Licor, Lincoln, NE). Detection of β-catenin, E-cadherin and Claudin-5 was performed using the primary antibody at 1:100 dilution followed by a secondary antibody conjugated to horseradish peroxidase and visualized by enhanced chemiluminescence (Thermo Scientific, Rockford, IL) [19]. All experiments were performed 3–5 times.

### Cell proliferation assay

The SNX27-WT and SNX27-KD MDA-MB-231 cells were plated at a density of 1 × 10^4^ cells per well in triplicate. After 24, 48 and 72 h of culture, the proliferation was evaluated by MTT Cell Proliferation Assay Kit (Thermo Fisher Scientific, Eugene, Oregon). Briefly, MDA-MB-231 cells were seeded in a 24-well plate at a density of 1 × 10^4^ cells/well and allowed to adhere before processing. After removing the medium, 500 μl per well of fresh culture medium without phenol red, and 50 μl of 12 mM 3-(4, 5-dimethylthiazol-2-yl)-2, 5-diphenyltetrazolium bromide (MTT) stock solution were added to the wells and the plate was incubated for 4 h at 37 °C in dark conditions. After adding 500 μl of the SDS-HCl solution prepared as the manufacture’s instruction, each well was mixed thoroughly using the pipette. Absorbance was measured using an enzyme-linked immunosorbent assay (ELISA) reader at 570 nm. Triplicate wells were counted for each time point and the whole experiment was repeated three times. The results were statistically analyzed using Welch’s t-test.

### Wound healing assay

Both WT and SNX27-KD clones of MBA-MD-231 cells were plated on fibronectin-coated 35 mm cover glass-bottomed dishes (MatTek, Ashland, MA) and cultured in high glucose Dulbecco’s modified Eagle’s medium (Invitrogen) supplemented with 10% FBS, 2.5 μg/ml of Puromycin and 1.5 μg/ml of doxycycline in humidified chambers with 5% CO_2_ at 37 °C. When cells were grown to confluency, the medium was aspirated, and the cell-coated surface was scraped with a 20-μl pipette tip in a single stripe. The scrape-wounded surface was washed twice with 37 °C warmed culture medium, and then the wounds in the cultures were allowed to heal for 24 h at 37 °C in a Olympus Vivaview incubator microscope (Olympus Optical Co., Germany) with 5% CO_2_. Migration of cells into wounded areas was captured at every 10 min for 24 h [20, 21]. The pictures were analyzed using a computer-assisted image analyzer (ImageJ, NIH, Bethesda, MD). The average extent of wound closure was evaluated by multiple measurements of area of the wound space in each of these cases. The migration rate was expressed as percentage of scratch closure and was calculated as follows: % of scratch closure = a-b/a, where (a) is an area between edges of the wound, and (b) is the area which remained cell-free during cell migration. The values are the means of three independent wound fields from three independent experiments.

### Matrigel 3D culture

WT or SNX27-KD clones of MBA-MD-231 cells were plated on Corning Costar Ultra-Low Attachment 96-well Plates and cultured in high glucose Dulbecco’s modified Eagle’s medium (Invitrogen, Carlsbad, CA) supplemented with 10% FBS, 2.5 μg/ml of puromycin and 1.5 μg/ml of doxycycline in humidified chambers with 5% CO_2_ at 37 °C for 24 h. Each cell cluster of SNX27-KD or WT was embedded in type I collagen gel solution (Cell-Matrix 3D cell culture kit, Nitta Gelatin Inc., Japan) at 5 × 10^4^ cells/ml. The collagen gels were incubated at 37 °C, 5% CO_2_ for 30 min until the gel became solid.

Cells in the collagen gels were cultured in DMEM with 10% FBS and 2.5 μg/ml of Puromycin and 1.5 μg/ml of doxycycline in humidified chambers with 5% CO_2_ at 37 °C for 24 h. To observe the 3D cell migration using nested collagen matrices, cultures were allowed to heal for 24 h at 37 °C Olympus Vivaview incubator microscope (Olympus Optical Co., Germany) with 5% CO_2_. Migration of cell clusters was captured at every 30 min for 48 h [20]. The pictures were analyzed using a computer-assisted image analyzer, ImageJ (Media Cybernetics, Bethesda, MD) [22]. The experiments were repeated at least three times.

### Assay for colony formation in soft agar

Cell invasion ability was determined by the colony formation assay in soft agar [23]. Both WT and SNX27-KD MAD-MB-231 cells (1 × 10^4^ cells/well) were mixed with 2 ml of selection media containing 10% FBS, 2.5 μg/ml of puromycin and 1.5 μg/ml of doxycycline and 0.5% agar (Sigma), and then overlaid on 2 ml of 0.8% agar in 6-well culture dishes (Corning Inc. NY). The cells were kept in humidified chambers with 5% CO_2_ at 37 °C for 2–3 weeks and the media were changed twice a week. After 2–3 weeks, colonies were visualized by staining with 0.05% crystal violet for 1 h. The number of colonies was then counted at four equal, randomly selected points in each well under phase contrast time-lapse microscopy (Primo Vert, Carl Zeiss, Oberkochen, Germany) at a magnification of 4 or 10 folds. The images were captured and processed using ImageJ software (Media Cybernetics, Bethesda, MD). The number of invaded cells for each experimental sample represents the average of triplicate wells [24]. All experiments were performed at least three times.

### Immunofluorescence and confocal imaging

WT and SNX27-KD MBA-MD-231 cells were plated on fibronectin-coated glass coverslips and cultured in high glucose Dulbecco’s modified Eagle’s medium (Invitrogen, Carlsbad, CA) supplemented with 10% fetal bovine serum, 2.5 μg/ml of puromycin and 1.5 μg/ml of doxycycline in humidified chambers with 5% CO_2_ at 37 °C. Cells were fixed with 4% paraformaldehyde for 10 min, permeabilized with 0.05% Triton X-100 for 5 min, washed with cold PBS twice, then incubated in blocking buffer (5% goat serum in PBS) for 1 h at room temperature. The fixed coverslips were incubated with primary antibodies diluted 1:100 in blocking buffer overnight at 4 °C. Coverslips were washed extensively with ice-cold PBS and incubated with the indicated secondary antibodies (Alexa Fluor 488, 568, 594, or 647 nm, Invitrogen) used at 1:100 dilution in blocking buffer for 1 h at room temperature. Following further washing with PBS, coverslips were mounted using FluorSaveTM (Calbiochem). DAPI was added (Life technologies) for 1 h at room temperature. Slides were mounted with SlowFade (Life technologies), followed by a cover slip, and the edges were sealed to prevent drying. Specimens were imaged using a Zeiss ApoTome 2 microscopy, a Leica TGS SP5 confocal microscope or a Nikon Eclipse TE300 microscope (Leica, Germany). All experiments were performed multiple times using independent biological replicates.

### Small interfering RNA (siRNA) treatment

SNX27-KD and WT MDA-MB-231 cells were plated in 12-well plates for 24 h before transfection. Cells were transfected with 25 nM E-cadherin siRNA (Santa Cruz Biotechnology), and control siRNA (Santa Cruz Biotechnology) using Lipofectamine-3000 transfection reagent (Invitrogen) according to the manufacturer’s instruction. The cells were harvested at 72 h post-transfection, and assayed by western blot.

### In vivo nude mice model

BALB/c Nude mice (*n* = 10) were purchased from Charles River Laboratories (Wilmington, MA,). These six to eight-week old, specific-pathogen-free, female mice were housed in Biologic Resources Laboratory (BRL) at University of Illinois at Chicago (UIC) and utilized in accordance with the UIC Animal Care Committee (ACC) and Office of Animal Care and Institutional Biosafety (OACIB) guidelines. The animals were subcutaneously injected bilaterally with 1.2 × 10^6^ cells in 200 μl of 50:50 Matrigel / PBS (phosphate buffered saline) into the hind flank [25]. At 16 days post-tumor challenge, mice were euthanized by IP injection of sodium pentobarbital (100 mg per kg body weight) followed by cervical dislocation, and tumors were collected and analyzed. The weight of the tumors was measured, and the tumor volume (V) was calculated from caliper measurements using previously published formulas V = (W^2^ × L) / 2 [26]. Fresh tumors were fixed in 10% neutral buffered formalin followed by paraffin embedding. For immunofluorescence staining (IF), slides were incubated in 5% BSA with 0.1% goat serum in PBS for 60 min at room temperature to reduce nonspecific background. The samples were incubated overnight at 4 °C with primary anti-E-cadherin, anti-β-catenin, and anti-Vimentin antibodies (Santa Cruz Biotechnology Inc.) at 1:100 dilution. The samples were then incubated with secondary antibodies and DAPI for 1 h at room temperature, and examined with confocal microscope as described before [27–29]. BrdU staining was performed as previously described [30]. Protein expression in tumor tissues was detected by western blot as described above.

### Statistical analysis

All the data in graphs were represented by the mean ± SD. All statistical tests were 2-sided. The *p*-values < 0.05 were considered statistically significant. All the *p*-values < 0.05, < 0.01 and < 0.001 were represented by one, two and three asterisks on the bars in the figures, respectively. Because Welch’s *t*-test is more reliable under the conditions of unequal variances and unequal sample sizes for the two-sample comparisons [31, 32], Welch’s *t*-test was utilized for all the two-group comparison. The differences among more than two groups were analyzed with One-way ANOVA using Turkey method to adjust multiple comparisons. The Welch’s *t*-test and One-way ANOVA were conducted with GraphPad Prism 5 (GraphPad Software, Inc. La Jolla, CA USA). To investigate the dynamic effects of SNX27-KD on cell migration comparing to the WT cells, the group effects from 24 to 72 h (Fig. 2A) and from 2 to 20 h (Fig. 2B) were tested using generalized linear mixed models. The statistical analyses were performed using SAS version 9.4 (SAS Institute, Inc., Cary, NC, USA).

## Results

### The expressional level of SNX27 in different breast cancer cell lines

**Fig. 1:**
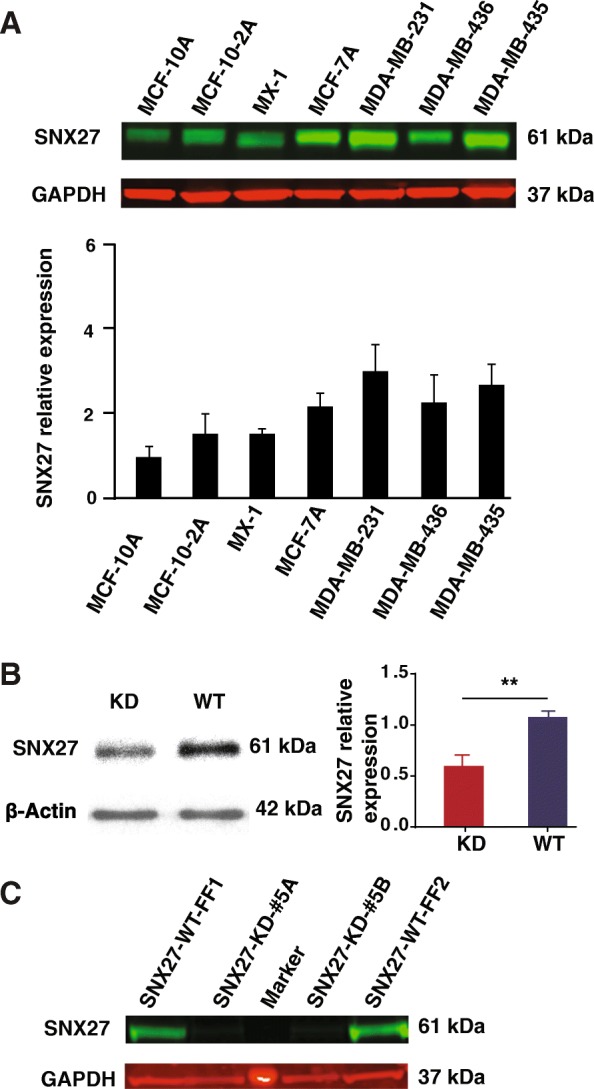
The SNX27 expression in multi-cell lines and knockdown in MDA-MB-231 cells. **a** Seven breast cancer cell lines were studied using western blot to detect the differences of SNX27 expression. The expression of SNX27 in MDA-MB-231 was the highest among the seven breast cancer cell lines, about three folds greater than MCF-10AH1’s expression. The relative protein level of SNX27 was quantified with measurement of band intensity and normalized on the MCF-10AH1. Data are analyzed with Welch’s t-test, and expressed as the mean ± SD from three independent experiments (*n* = 3). **b** and **c** Western blot showed that stable SNX27-KD MDA-MB-231 cells were successfully generated using an inducible lentiviral shRNA system (pINDUCER10) after treating for 48-h (**b**) and 72–96 h (**c**). Data are analyzed with Welch’s t-test, and expressed as the mean ± SD from three independent experiments (n = 3). **p < 0.01 vs WT control. KD: knockdown of SNX27 in MDA-MB-231 cell; WT: wildtype of MDA-MB-231 cell

In order to compare the difference in expression of SNX27 in different malignant breast cancer cell lines, we collected 7 breast cancer cell lines with different levels of aggression. As these breast cancer cell lines are derived from clinical tumors with various features, the categorization, molecular information, protein expression and invasiveness are also different. For instance, MDA-MB-231 cells are invasive while MCF-7 cells are non-invasive. Meanwhile, MCF-10AH1 cells are the least aggressive cancer cells [33]. Equal amounts of cell lysis were analyzed by western blot. The results showed that SNX27 expression in MDA-MB-231 was the highest level among the seven aggressive breast cancer cell lines, about three folds greater than MCF-10AH1’s expression (Fig. 1a).

### Establishment of the SNX27 knock-down (KD) clone in MDA-MB-231 cells

To investigate the effects of SNX27 on tumor cell growth, we generated an inducible lentiviral-mediated shRNA expression system to suppress the expression of SNX27 in MDA-MB-231 cells, which showed the highest SNX27 translational level as described above. Puromycin-resistant clones were treated with doxycycline to induce shRNA expression. Expression of the red fluorescent protein marker indicative of shRNA expression could be detected 48 h post-induction; at this point expression of SNX27 remained but was reduced in the SNX27 KD cells compared to the WT control *(p* < 0.01) (Fig. 1b). After 72–96 h treatment, the expression level of SNX27 was below the threshold of detection (Fig. 1c) and about 90% of SNX27 expression was suppressed in KD cells compared with mock transfection. The reduced SNX27 expression in the clones was persistent which indicated that we established a stable SNX27 knockdown in MDA-MB-231 cells.

### SNX27 knockdown reduced cell proliferation and cell migration

**Fig. 2:**
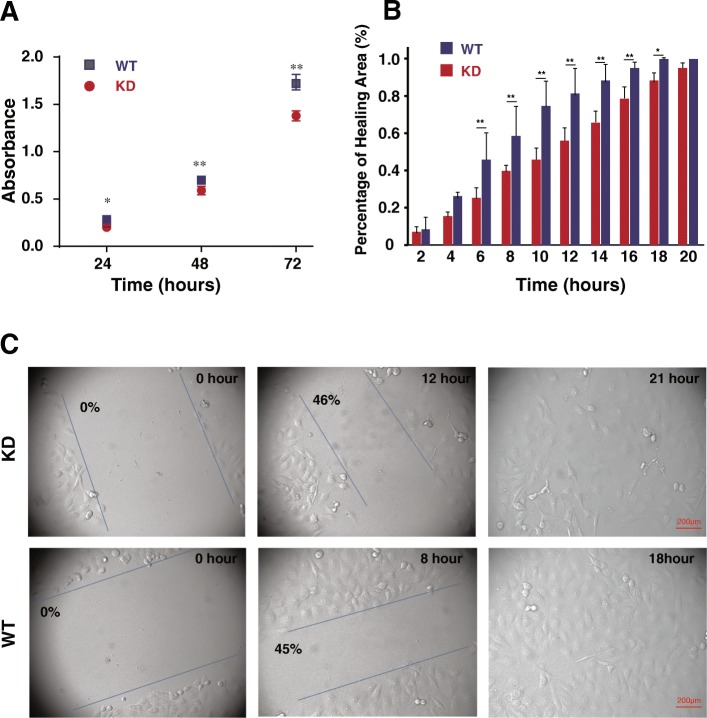
Knockdown of SNX27 reduced the proliferation and migration of MDA-MB-231 breast cancer cells. **a** Proliferation of SNX27-KD MDA-MB-231 cells was significantly decreased comparing with WT control MDA-MB-231 cells by MTT assay conducted at time points 24, 48, and 72 h. Triplicate samples of each wafer from three independent experiments were included at each of the 3 time points (*n* = 3). Data are analyzed using generalized linear mixed models, and expressed as mean ± SD. *p < 0.05 vs. WT control; **p < 0.01 vs. WT control. **b** and **c** Wound healing analysis of SNX27-KD and WT MDA-MB-231 cells showed that the WT cancer cells can recover ~ 50% of the wound area in 8 h and heal the whole area in 18 h, but KD cells took 3 h longer than WT cancer cells to cover the whole wound area. Meanwhile, the percentages of healing area were showed with different time points. The migration rate was expressed as a percentage of scratch closure. The values are the means of three independent wound fields in three repeated time. Data are analyzed using generalized linear mixed models, and expressed as mean ± SD from three independent experiments (*n* = 3). *p < 0.05 vs. WT control; **p < 0.01 vs. WT control. Scale bar is 200 μm

After establishing the SNX27-KD clone in MDA-MB-231 cells, we then examined the cell proliferation of SNX27-KD cells by the MTT assay, which is a colorimetric assay for assessing viable cell metabolic activity. Our data showed that cell proliferation was significantly reduced in SNX-KD cells after 24–72 h incubation (*p* < 0.01) (Fig. 2a), indicating that the expression level of SNX27 is important in regulating cell proliferation.

To investigate the role of SNX27 in cell migration, we conducted a wound-healing experiment. Our data showed that SNX27-KD cells healed the wound area significantly slower than the WT cells (*p* < 0.01) (Fig. 2b). It took 12 h and 21 h for KD cells to cover the 50 and 100% of wound area, respectively, whereas the WT could heal the 50% of wound area within 8 h and 100% of the area in 16–18 h (Fig. 2c). Statistic analysis showed that SNX27 knockdown significantly reduced cell migration starting from 6 h post wound healing (Fig. 2b).

### SNX27 KD changed colony formation in soft agar and altered EMT regulators

**Fig. 3:**
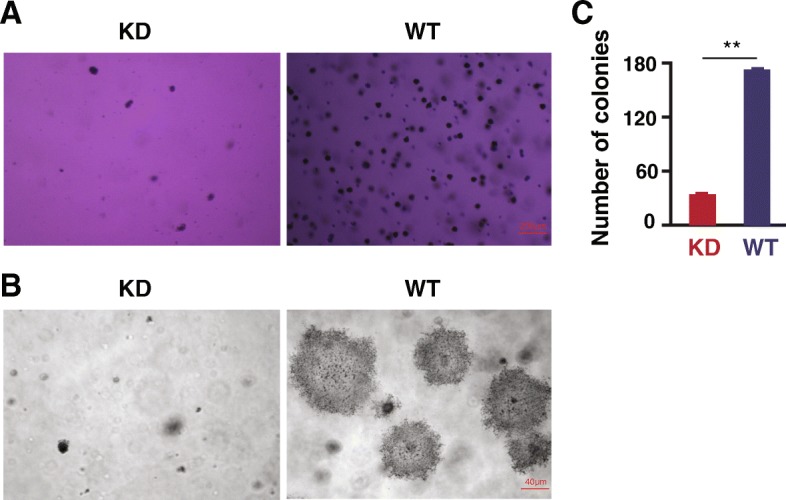
The aggressiveness of SNX27-KD and WT MDA-MB-231 cells in soft agar. To investigate the influence of SNX27 on the aggressiveness of breast cancer MDA-MB-231 cells, the colony forming ability of SNX27-KD and WT MDA-MB-231 breast cancer cells, were observed by soft agar assays. The pictures taken under an inverted phase-contrast microscope with 4× (**a**) 20× (**b**) magnifier object. **c** SNX27 knockdown cells formed significantly fewer colonies than the parental MDA-MB-231 cells. Data are analyzed with Welch’s t-test, and expressed as mean ± SD from three independent experiments (n = 3). **p < 0.01 vs. WT control

Colony formation is a hallmark of cancer cell malignancy in vitro [24]. As shown in Fig. 3, knockdown of SNX27 significantly suppressed the colony formative capability of MDA-MB-231 cells (Fig. 3a). The colony size of SNX27 KD was smaller than that in the WT cells (Fig. 3b). We also counted the colony numbers. We found that the colony numbers of SNX-27-KD MDA-MB-231 cells were statistically fewer compared to those from the WT cells (SNX27 KD vs. WT, *p* < 0.01) (Fig. 3c).

**Fig. 4:**
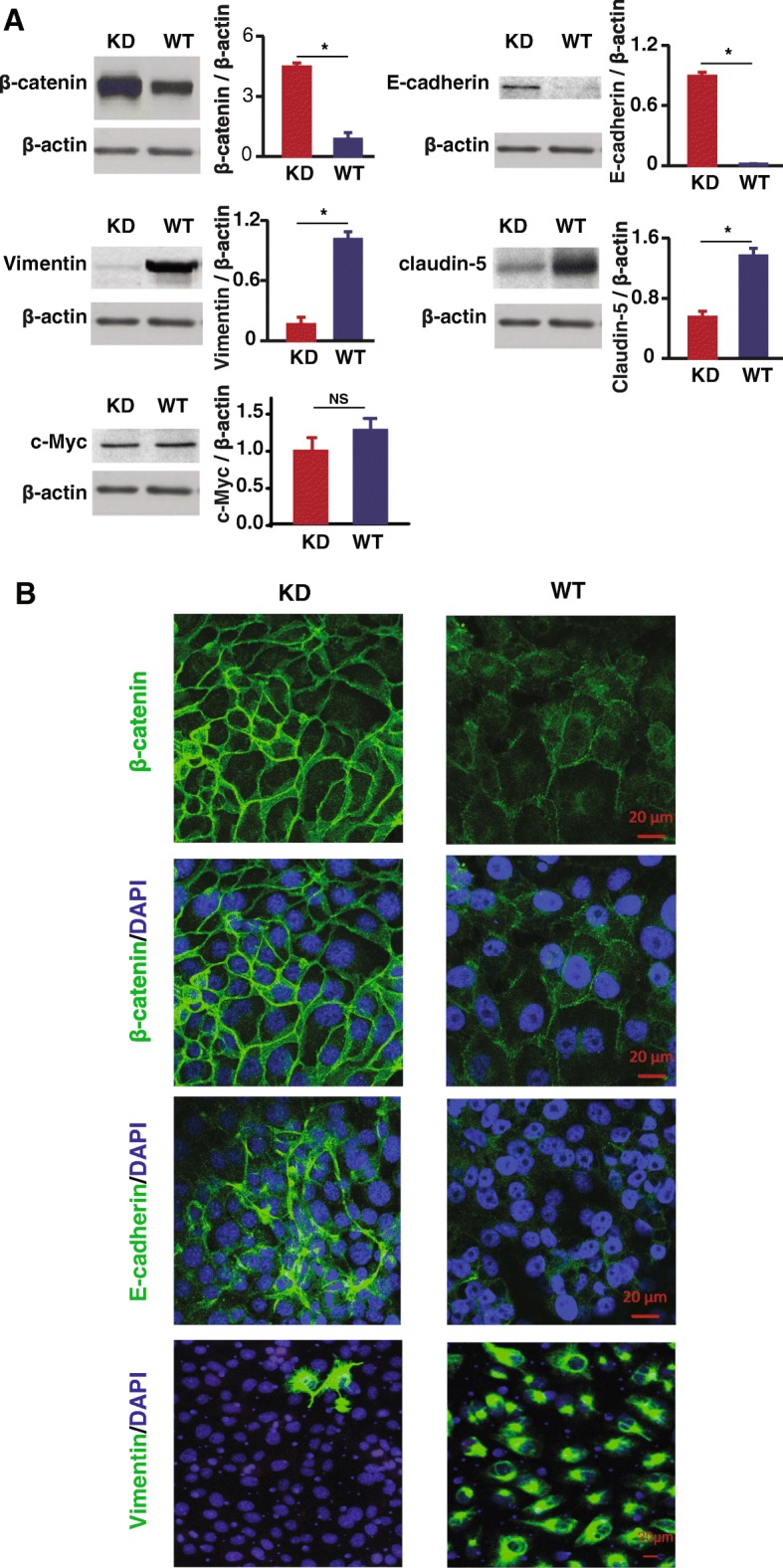
Expression of critical transcription factors of EMT in SNX27-KD and WT MDA-MB-231 breast cancer cells. **a** Western blots showed the protein expression of β-catenin and E-cadherin was significantly increased after SNX27 knockdown, whereas the expression of Vimentin and Claudin-5 were significantly reduced. However, there is no significantly difference of some other cell proliferation markers, like c-Myc, between KD and WT cells. Data are analyzed with Welch’s t-test, and expressed with mean ± SD from three independent experiments (n = 3). *p < 0.05 vs. WT control; NS: no significantly difference vs. WT control. **b** SNX27-KD and WT MDA-MB-231 cells were immunostained with β-catenin, E-cadherin, and Vimentin, respectively. Knockdown of SNX27 led to enhanced β-catenin in cell membrane, which is involved in extracellular matrix formation with E-cadherin. The confocal imaging also confirmed that Vimentin is expressed at extremely low levels after SNX27 knockdown (Scale bar is 20 μm). Duplicate samples were included in three independent experiments (n = 3)

To elucidate the mechanism of how knocking down SNX27 leads to suppressed cancer cell proliferation and migration, we investigated the expression of EMT marker proteins. Our data showed that SNX27-KD significantly increased expression of β-catenin and E-cadherin, whereas the EMT regulatory marker Vimentin was significantly suppressed (Fig. 4a). As the major cytoskeletal component of mesenchymal cells, Vimentin is often used as a marker of mesenchymally-derived cells or cell-undergoing EMT. Here we found that Vimentin expression was suppressed after knockdown of SNX27 in MDA-MB-231 cells. Claudin-5 is a tight junction protein, which is highly expression in breast cancer patient with the high-risk metastasis and reoccurrence [34]. SNX27-KD cells showed significantly lower Claudin-5 expression in western blots (Fig. 4a). However, there was no significant difference of c-Myc expression between SNX27-KD and WT MDA-MB-231 cells. c-Myc is often constitutively expressed in cancer, which leads to increased expression of many genes, including cell proliferation related genes. Immunostaining data also showed the same trend of EMT-related protein expression in MDA-MB-231 breast cancer cells (Fig. 4b).

**Fig. 5:**
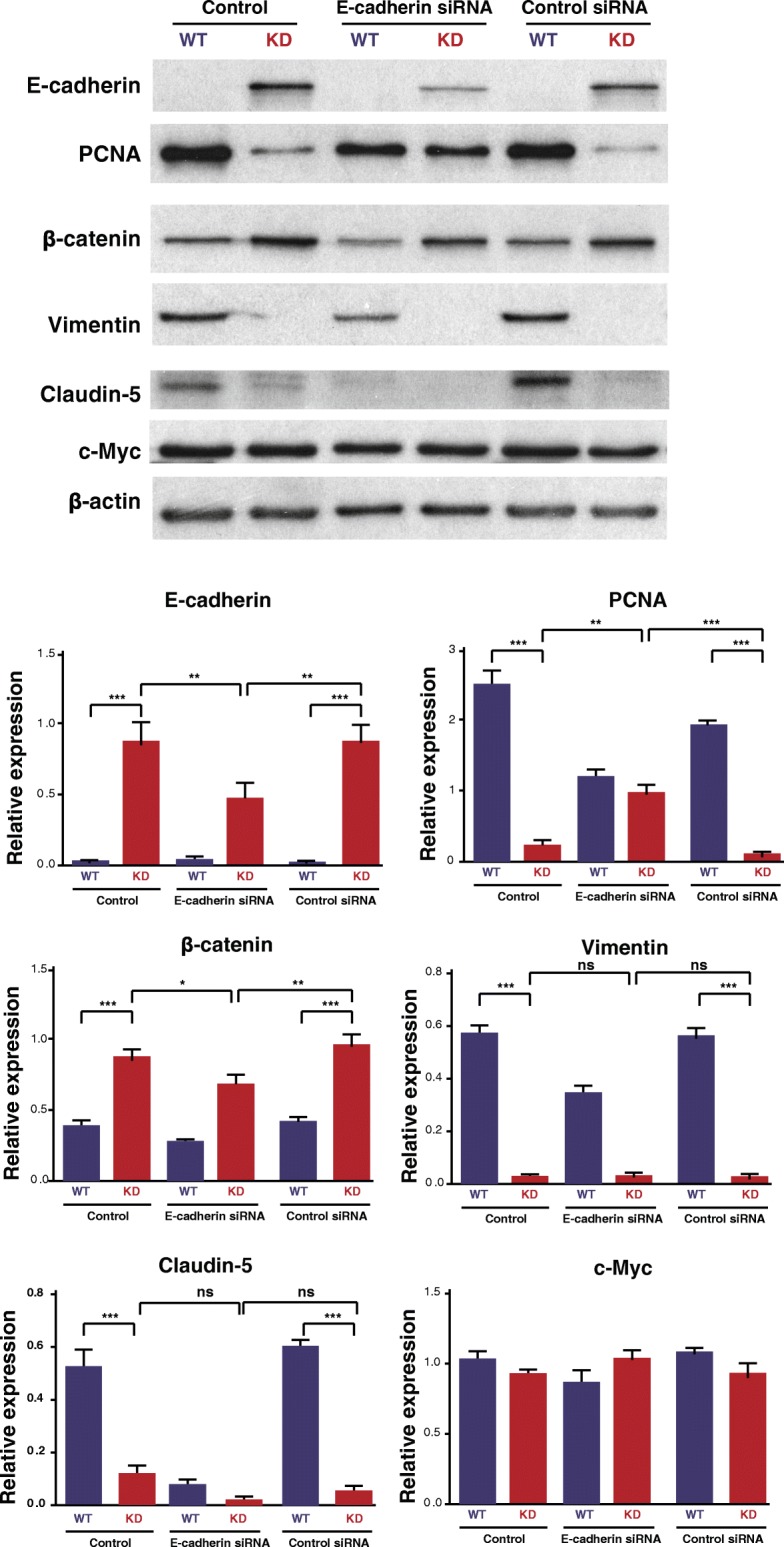
Confirmation the effect of SNX27 knockdown on E-cadherin activity by siRNA transfection. SNX27-KD and WT cells (50–60% confluence in 12-well plates) were transfected with E-cadherin siRNA. Lysates were blotted with anti-E-cadherin, anti-PCNA and anti-EMT-related markers. The SNX27-KD MDA-MB-231 cells transfected with E-cadherin siRNA were significantly decreased the E-cadherin expression compared with cells treated with control siRNA and transfection reagent control. The expression of PCNA, the cell proliferation marker, was increased in SNX27-KD cells transfected with E-cadherin siRNA. Data are analyzed with One-way ANOVA adjusting multiple comparisons with Turkey method, and expressed with mean ± SD from six independent experiments (*n* = 6). ns: no significantly difference, *p < 0.05, **p < 0.01, ***p < 0.001

We further blocked the expression level of E-cadherin in SNX27-KD and WT MDA-MB-231 cells to see whether there were any influences on cell proliferation and junctions. The expression of cell proliferation marker (PCNA) was increased after E-cadherin siRNA transfection, which indicated that the cell proliferation phenotype of SNX27-KD cells could be partially reversed (Fig. 5). E-cadherin siRNA in SNX 27 KD MDA-MB-231 breast cancer cells also reduced β-catenin, which is known to bind with E-cadherin in cell junctions, but did not significantly affect tight junction protein Claudin5 or proliferation regulator c-myc. The WT cells already have very low level of E-cadherin protein, which is untestable by western blots reversed (Fig. 5a). The reduced PCNA in WT cells was observed in both E-cadherin siRNA group and Control siRNA group. It indicated that the reduced PCNA in WT cells is not specifically due to the E-cadherin siRNA.

### Impact of SNX27 knockdown on tumor growth and EMT regulators in vivo

**Fig. 6:**
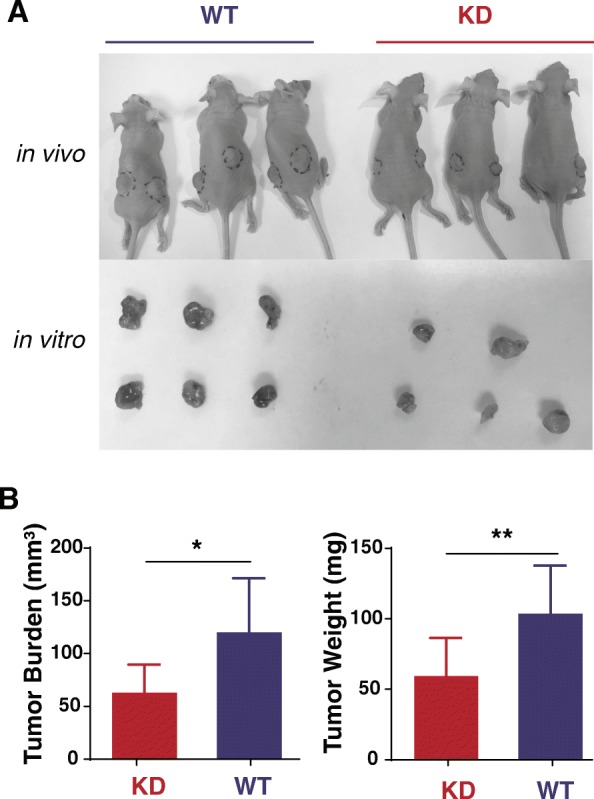
Breast cancer of SNX27-KD MDA-MB-231 cells grew less than that of WT MDA-MB-231 cells in a nude mouse model. **a** Representative tumors harvested from the mice (*n* = 5 per group) which challenged with SNX27-KD and WT MDA-MB-231 breast cancer cells were showed in vivo and in vitro. **b** Volume and weight of tumors (*n* = 9 per group) harvested from the mice injected with SNX27-KD MDA-MB-231 cells was significantly lower than that of wildtype cancer cells. Data are analyzed with Welch’s t-test, and expressed with mean ± SD. *p < 0.05 vs. WT control, **p < 0.01 vs. WT control

Direct orthotopic or heterotopic injection of breast cancer cells into the mouse have been used to replicate the changes of cells in vitro [35–41]. These traditionally used xenograft models allow the proliferation of human tumor cells in the mouse model. To investigate the impact of SNX27 knockdown on tumor outgrowth, we adapted the breast cancer xenograft nude mouse model by injecting SNX27-KD or WT MDA-MB-231 cells. We found that tumors were significantly larger and heavier in mice injected with the wildtype MDA-MB-231 cells (*n* = 5), compared to mice injected with SNX27-KD MDA-MB-231 cells (n = 5) (*p* < 0.05) (Fig. 6).

**Fig. 7:**
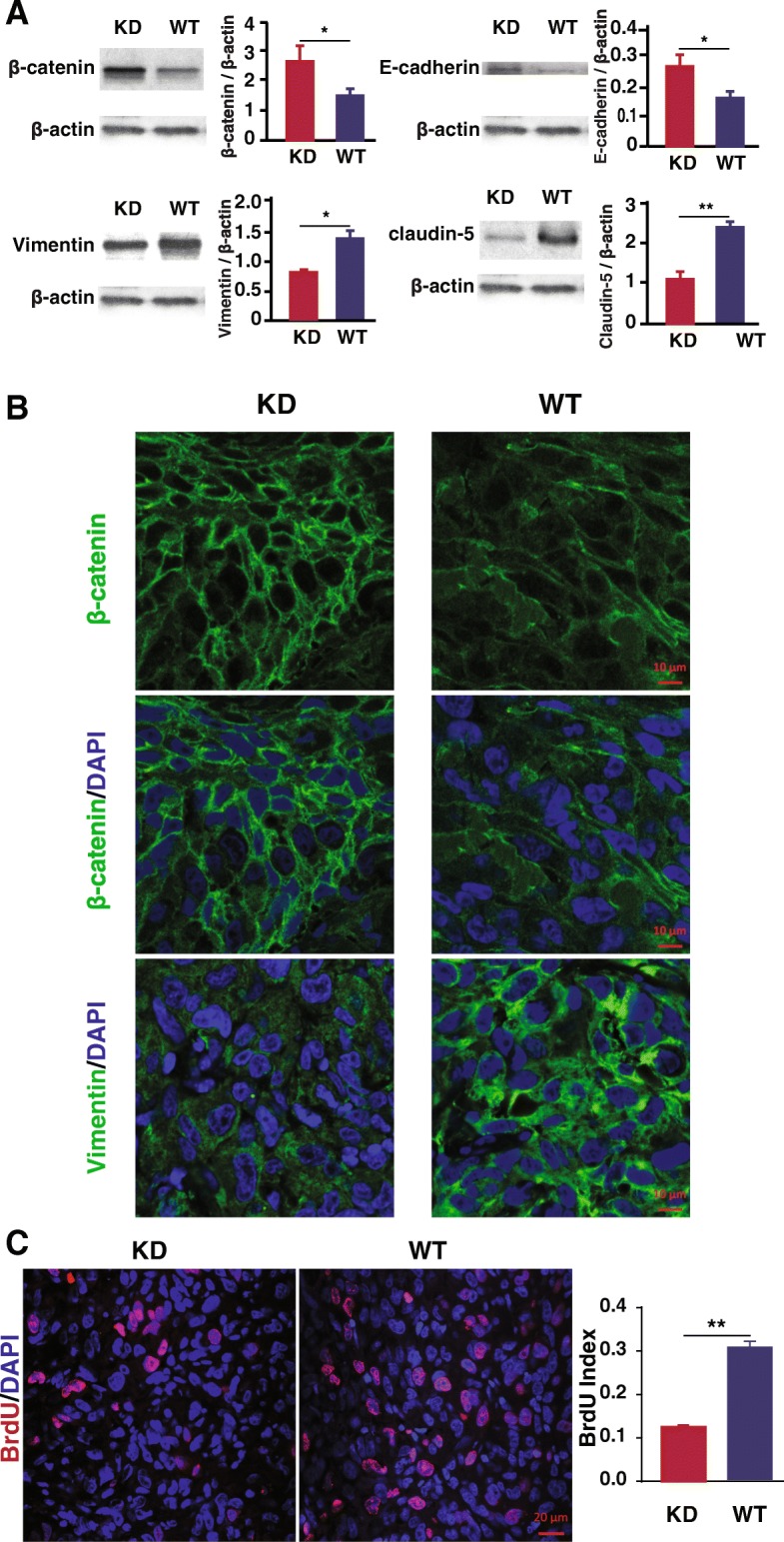
Expression of critical EMT regulators in tumors from nude mice. **a** SNX27-KD MDA-MB-231 cells accelerated the protein expression of E-cadherin and β-catenin, and decelerated the protein expression of Vimentin and Claudin-5 in the tumors isolated from the nude mice model injected with breast cancer cells. Data are analyzed with Welch’s t-test, and expressed with mean ± SD from three independent experiments (n = 3). *p < 0.05 vs. WT control. **p < 0.01 vs. WT control. **b** Immunofluorescence staining of β-catenin and Vimentin was shown that these proteins were expressed in tumors from SNX27-KD injected mice and WT MDA-MB-231 breast cancer cells. Scale bar is 10 μm. Duplicate samples of each wafer were included in three independent experiments (n = 3). **c** Cell proliferation in the tumor tissue of mice injected with SNX27-KD cells were decelerated compared with wildtype cells by immunofluorescence staining of BrdU (scale bar is 20 μm). BrdU index (number of cell stained with BrdU / number of total cell) was significantly higher in mice injected with wildtype MD-MB-231 cells compared with SNX27-KD cells. Duplicate samples of each wafer were included in three independent experiments (n = 3). **p < 0.01 vs. WT control

We then tested the markers of EMT and tight junction proteins in the tumors. Western blot analysis showed that the SNX27-KD-induced tumors had significantly increased E-cadherin and β-catenin, but ablated expression levels of Vimentin and Claudin-5 (Fig. 7a). Immunofluorescence staining showed increased β-catenin and reduced Vimentin in SNX27-KD injected mice, compared to that in WT MDA-MB-231 breast cancer cells (Fig. 7b). Furthermore, proliferation assay of BrdU index (number of cell stained with BrdU / number of total cell) was significantly higher in mice injected with WT MD-MB-231 cells, compared with mice injected with SNX27-KD cells (Fig. 7c), suggesting more cell growth in the WT breast cancer cells in vivo.

## Discussion

**Fig. 8:**
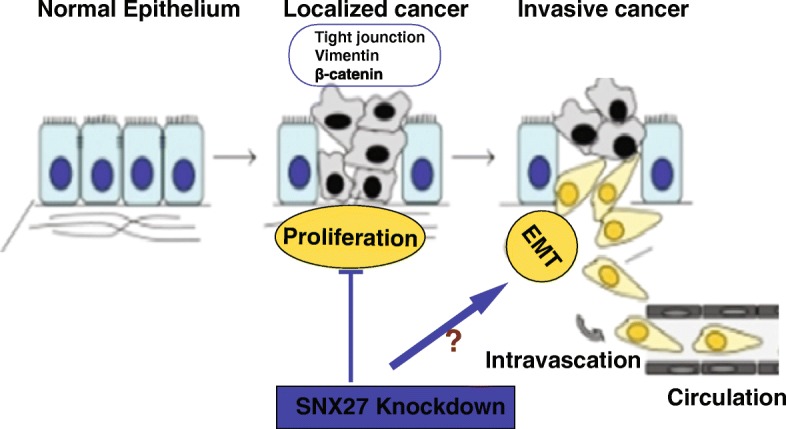
The working model of SNX27 knockdown in the cancer development. Knockdown of SNX27 inhibits the proliferation and decreases the expression of Vimentin and Claudin-5, and increases the expression of E-cadherin and β-catenin, which impact on the cell adhesion and migrate. SNX27 could be considered a potential molecular target for controlling cancer growth and development

Here, we have demonstrated that SNX27 plays a novel and crucial role in breast tumor cell growth. Knockdown of SNX27 leads to expression changes of E-cadherin, β-catenin and Claudin-5, which facilitate adhesion formation, and reduction of Vimentin, which is a critical transcription factor in EMT. The proliferation and collective migration of SNX27-KD MDA-MB-231 cancer cells were significantly reduced. The suppressed proliferation can be reversed in the SNX27-KD cells with increasing PCNA, the cell proliferation marker, using E-cadherin siRNA transfection (Fig. 5). These differences were further confirmed in the nude mouse model in vivo. Overall, knockdown of SNX27 reduced the growth of MDA-MB-231 breast cancer cells (Fig. 8).

To elucidate the mechanism of how SNX27 knockdown impacted the tumor cell behavior and phenotype, we assessed the effect of SNX27 knockdown on biomarkers of EMT. In our study, knockdown of SNX27 markedly increased the expression of E-cadherin and β-catenin. Confocal imaging also detected E-cadherin and β-catenin location mainly at the cell membranes after SNX27 knockdown, indicating increased cell stability. The location of β-catenin at the cell membrane in the SNX27 KD cells suggests increased cell adherence, rather than cell proliferation which marked by nuclear translocation of β-catenin. EMT is a cellular program that allows polarized, immotile epithelial cells to convert to motile mesenchymal cells, enabling carcinoma invasion and metastasis. Controlling or blocking EMT processes is a promising target in developing anti-cancer drugs^,^ [42–44]. EMT is dynamically controlled by various proteins [45–50]. The role of SNX27 in inhibiting EMT should be further studied in vivo in future research.

The importance of SNX27 in breast cancer development was confirmed in vivo with the xenograft nude mouse model. Our data indicated that SNX27 may play a crucial role in cancer cell proliferation, which is consistent with previous reports that SNX27 is required for HeLa and mpkCCD cell migration [17]. Here, the up-regulation of E-cadherin and β-catenin, and down-regulation of Vimentin and Claudin-5 was detected in the nude mice after injecting SNX27-KD MDA-MB-231 cells. Our results suggest the SNX27 regulates E-cadherin, one of the tumor suppressors, thus decreasing cell proliferation.

In the current study, we also detected that knockdown of SNX27 significantly suppressed the expression of Vimentin in breast cancer cells. The dramatic reduction of Vimentin in SNX27 KD cells is very interesting. Vimentin connects mechanotransduction webs within cells, from focal adhesions to cytoskeletal proteins, such as actin, microtubules, and nuclear cytoskeleton nesprins, to modulate cell spreading and adhesion, and control the directional migration of cancer cells [51]. Many basic and clinical studies have found Vimentin to be a poor prognostic biomarker for breast cancer [52, 53]. Knockdown of Vimentin can also result in the lower expression of Slug and reduce cell migration and proliferation [51]. Thus, the SNX27-related depletion of Vimentin may further stabilize the cytoskeletal architecture within cells to reduce cell motility. SNX27 may mainly affect tumor cell EMT by altering the expression of EMT-regulating proteins, thus reducing the tumor growth and migratory properties.

Interestingly, we found that SNX27 KD could significantly reduce tight junction protein Claudin-5 expression. Claudins are major adhesion molecules in tight junctions (TJs) and are strongly expressed in various cancers [54]. Claudin-5 has been found to be highly expressed in breast cancer patients with high-risk metastasis and reoccurrence [34]. SNX27 KD significantly reduced the amount of Claudin-5 and decreased the cell-to-cell tight junctions that made the tumor cells “glue” together. SNX27 contains a PSD95, Dlg1, ZO-1 (PDZ)-binding motif which promotes recycling of internalized transmembrane proteins from endosomes to the plasma membrane. A proteomics study has indicated that interactions between SNX27 and ZO-2 play a key role in tight junction maintenance and function [3]. Further studies are needed to understand the mechanisms of how SNX27 interacts with TJ proteins, including Claudin-5.

One limitation of the current study is that we did not have SNX27 completely knockout in the MDA-MB-231 cells. We wound expect a greater effect on cellular proliferation and the EMT pathway after complete knockout of the SNX27 from the breast cancer cells. Also, we used the xenograft model by injecting the cells in the back flank of nude mice, which does not reflect the real development of breast cancer occurring in the mammary gland. The role of SNX27 should be further studied in a chemical-induced or a transgenic breast cancer model. In the future, we will also plan to determine the role of SNX27 in other cancer cells.

## Conclusions

In conclusion, we have demonstrated a novel function for SNX27 in regulating the growth of cancer cells. Collectively, knockdown of SNX27 markedly suppressed the proliferation, migration and colony formation ability of breast cancer cells. The knockdown of SNX27 resulted in a loss of aggressiveness of cancer cells in vitro and in vivo. SNX27 plays a crucial role in tumor progression. Our findings provide insights into manipulating SNX27 as a new target gene to control tumor growth and development.

## Data Availability

The datasets used and analyzed during the current study are available from the corresponding author on reasonable request.

## Abbreviations

ELISA: Enzyme-linked immunosorbent assay
EMT: Epithelial-mesenchymal transition
GLUT1: Glucose transporter 1
IF: Immunofluorescence
KD: Knockdown
MTT: 3-(4,5-Dimethylthiazol-2-yl)-2,5-Diphenyltetrazolium Bromide
PDZ: PSD95, Dlg1, ZO-1
PX: Phox-homology
SNX27: Sorting Nexin 27
TJ: Tight junction
WT: Wild type
